# Supplementary figures and images for: Microbial communities can predict the ecological condition of headwater streams
Source: PLoS One. 2020 Aug 3;15(8):e0236932. doi: 10.1371/journal.pone.0236932 (PMC7398514; doi:10.1371/journal.pone.0236932)

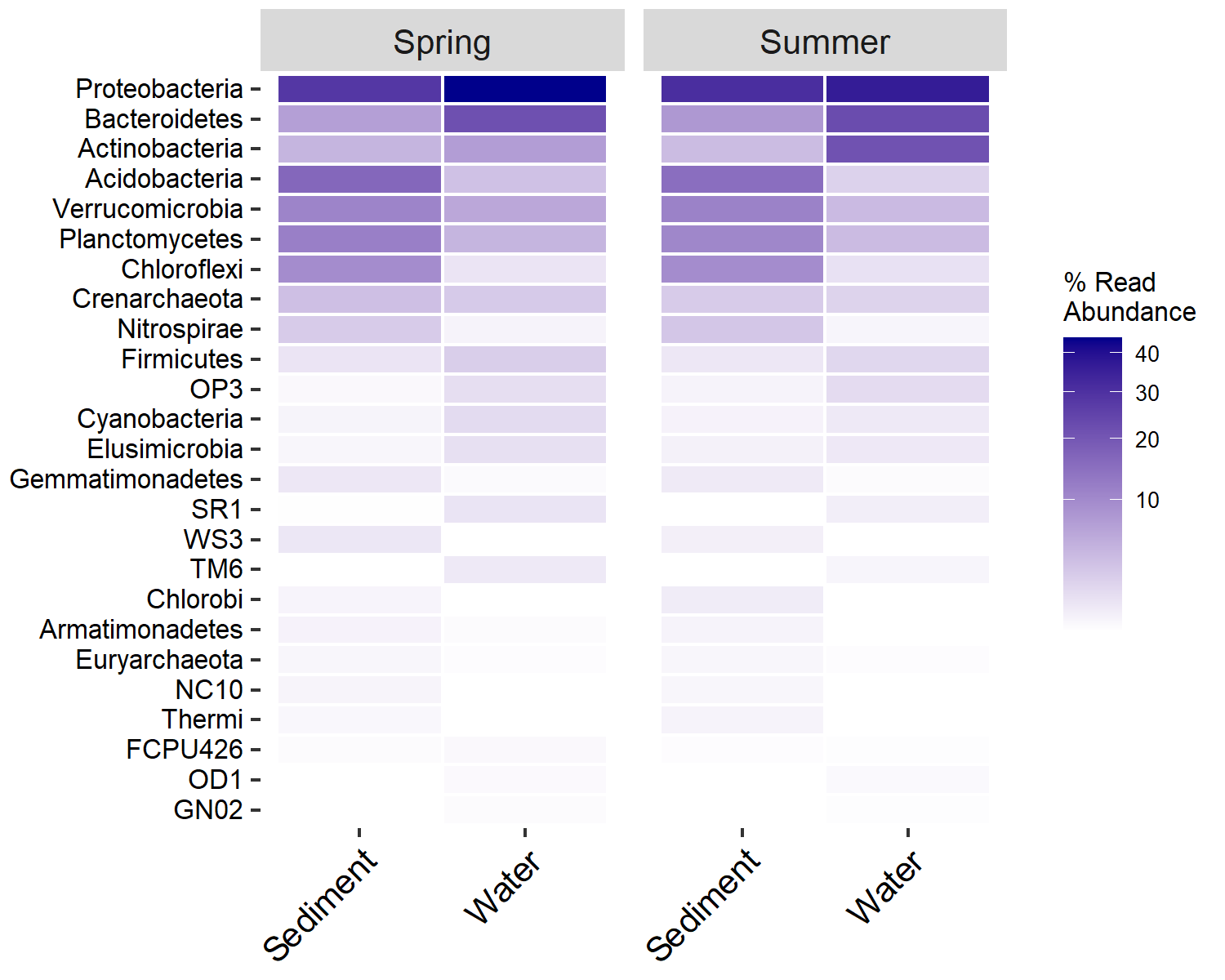

Supplement: S1 Fig — (TIFF) [file pone.0236932.s001.tiff]

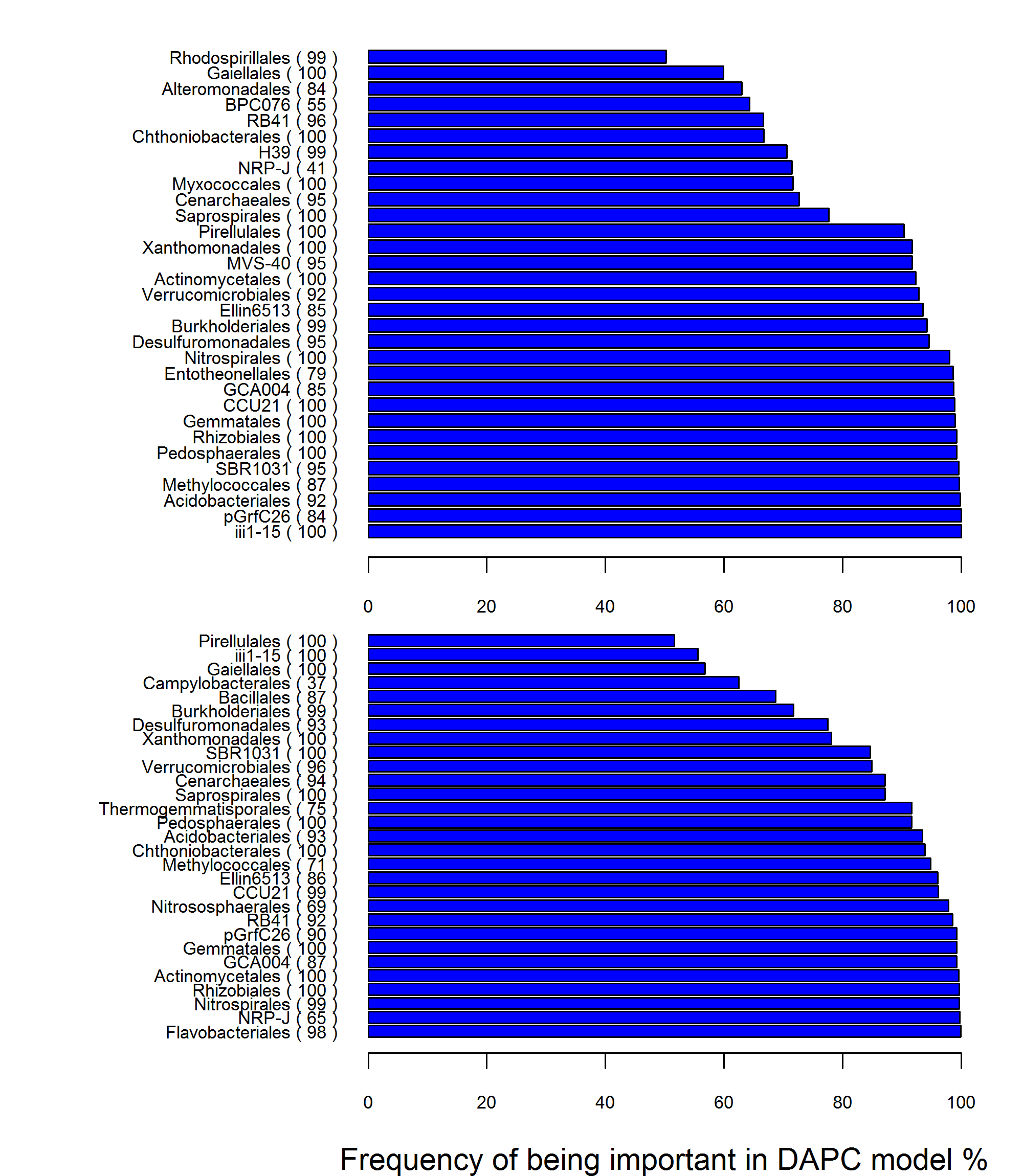

Supplement: S2 Fig — Frequencies of inclusion in the DAPC analysis as an important order for the most important order-level taxa to predicting stream condition from summer sediment (top panel) and spring sediment (bottom panel) samples. The numbers in parentheses beside each taxon indicate its relative frequency of occurrence at sites in spring or summer sediment samples. (TIFF) [file pone.0236932.s002.tiff]

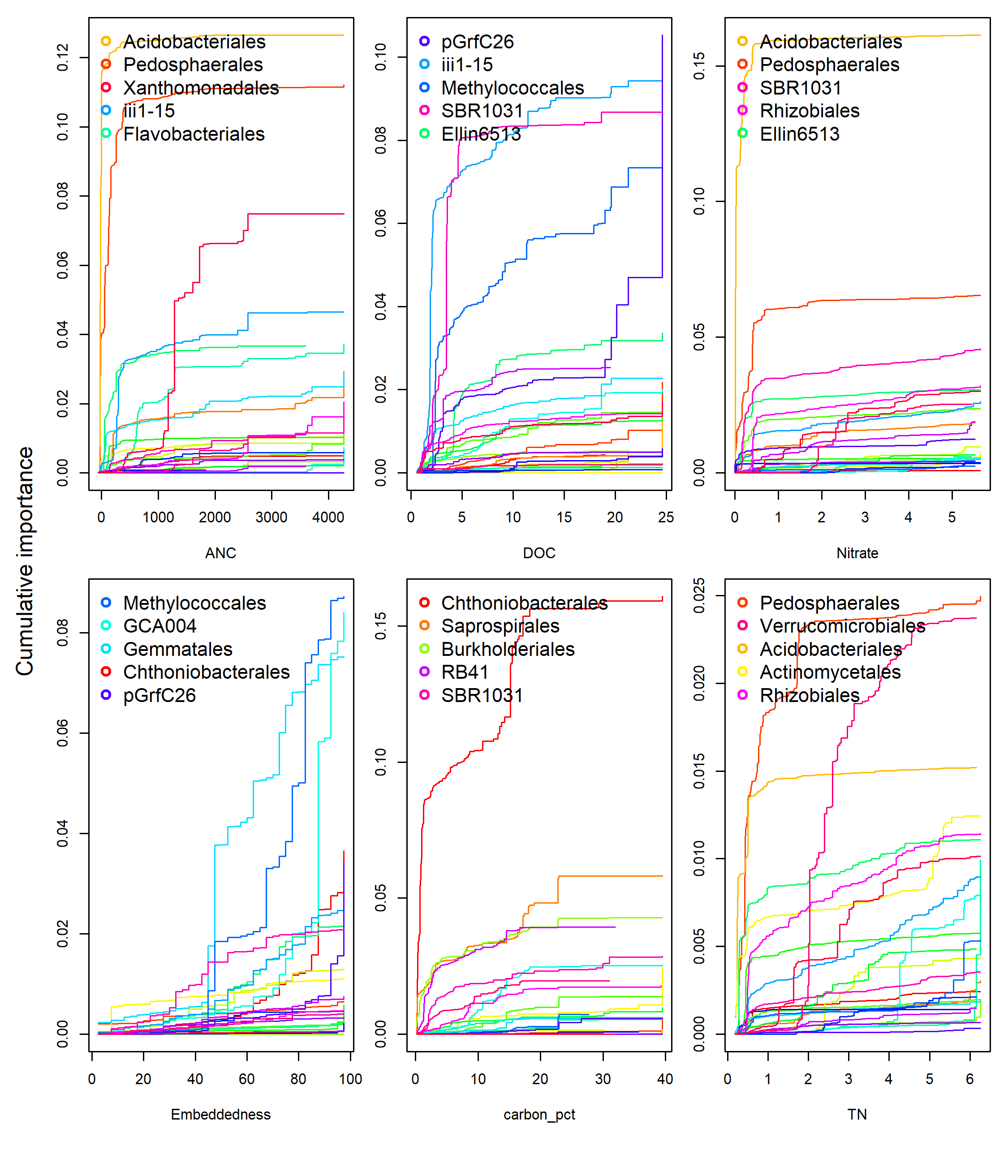

Supplement: S3 Fig — (TIFF) [file pone.0236932.s003.tiff]

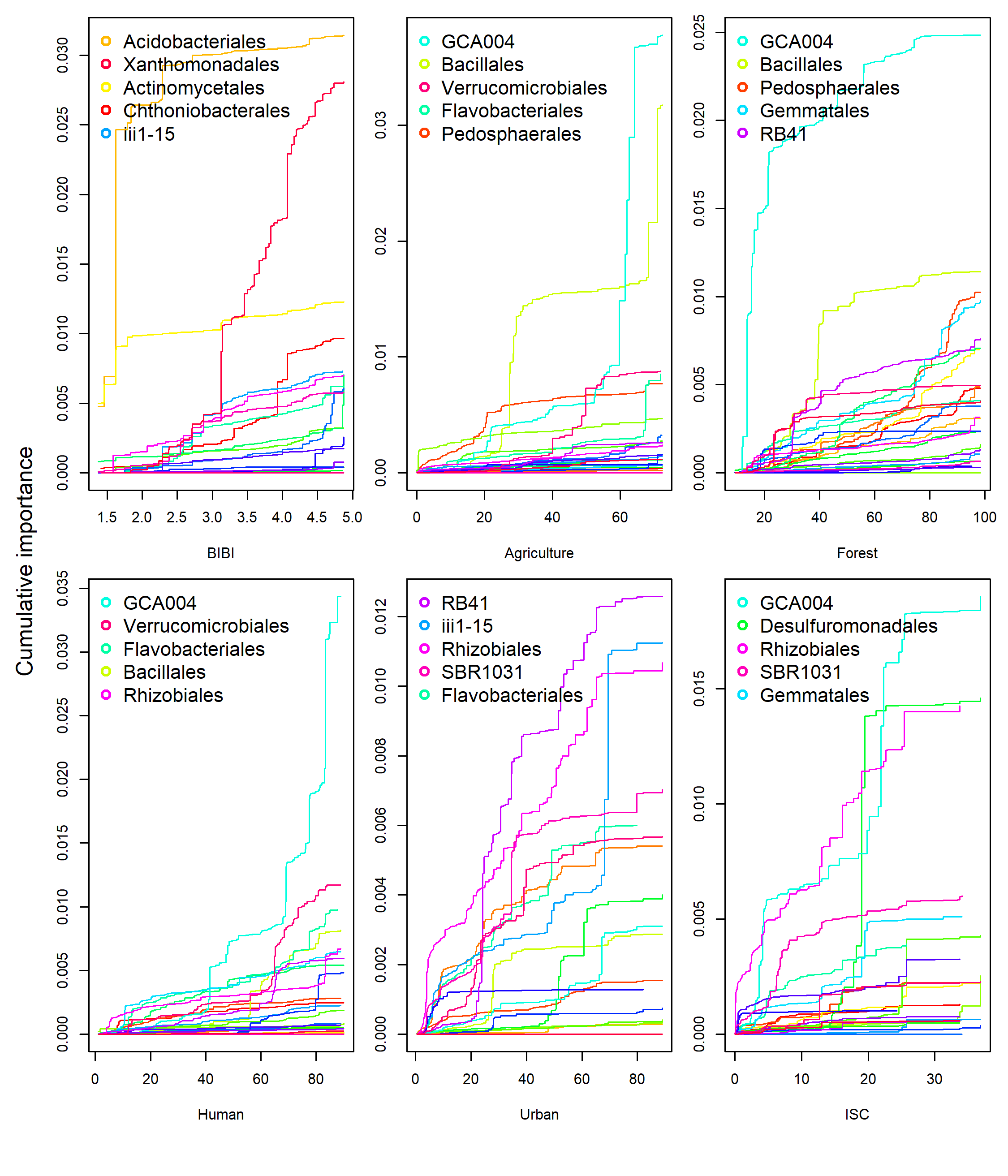

Supplement: S4 Fig — (TIFF) [file pone.0236932.s004.tiff]

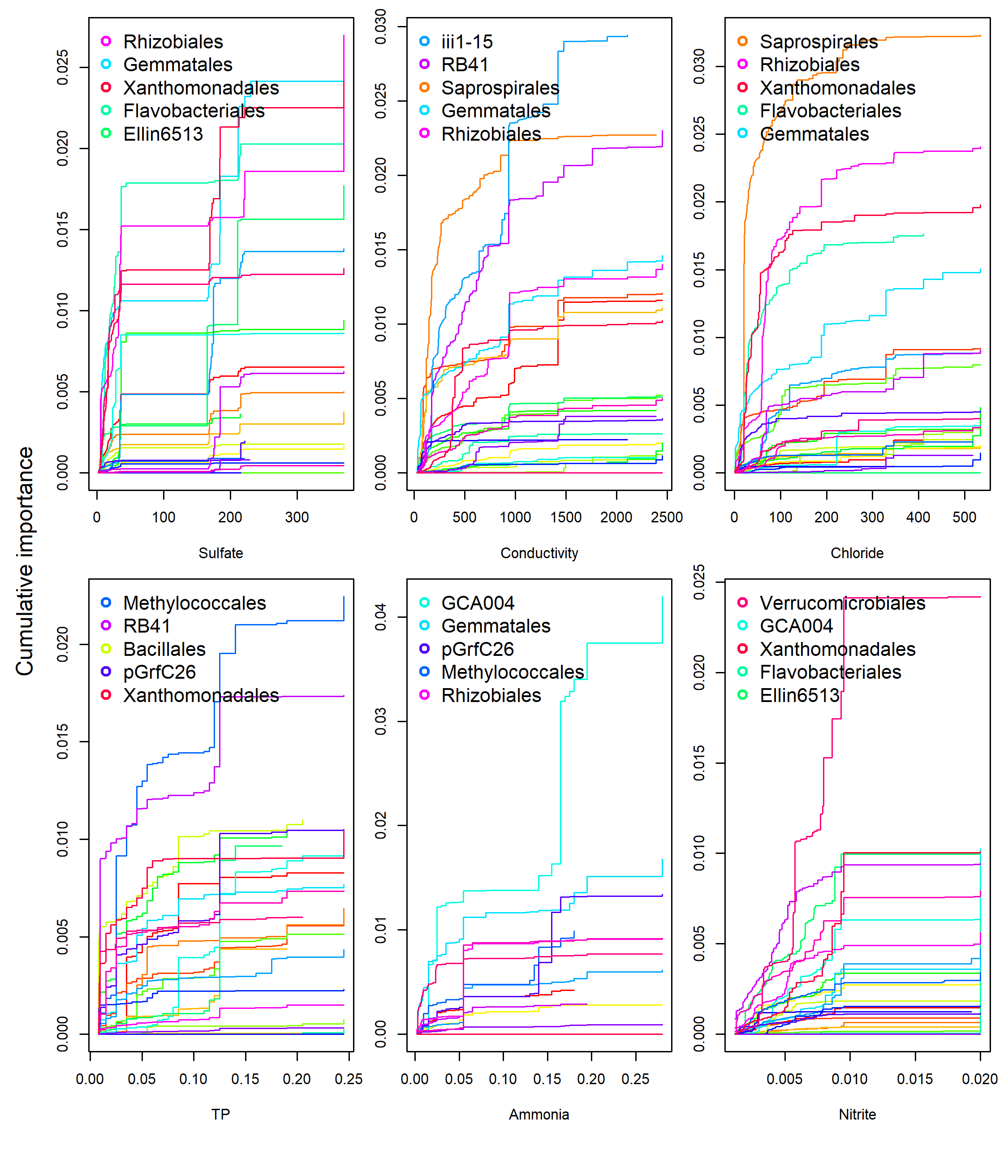

Supplement: S5 Fig — (TIFF) [file pone.0236932.s005.tiff]
