# Supplementary material for: Microbial communities can predict the ecological condition of headwater streams
Source: PLoS One. 2020 Aug 3;15(8):e0236932. doi: 10.1371/journal.pone.0236932 (PMC7398514; doi:10.1371/journal.pone.0236932)
Supplement: Attachment — Submitted filename: Response to Reviewers.docx [file pone.0236932.s006.docx]

I believe that I have addressed every one of the comments and have rarely rebutted. I have included the review comments below, and I have highlighted my responses in yellow as well as indicated where those changes occur in the revision in **bold**.

Please note that I have slightly revised the title of the manuscript to better reflect its contents.

I hope that my revision is suitable for acceptance and publication in PLOS ONE. Thank you. bob

####################

Thank you for submitting your manuscript to PLOS ONE. After careful consideration, we feel that it has merit but does not fully meet PLOS ONE’s publication criteria as it currently stands. Therefore, we invite you to submit a revised version of the manuscript that addresses all the points raised by the reviewer. In agreement with this reviewer, I was also very surprized to see that you have pooled your triplicate samples, depriving you of important data. I share also the same astonishment than the reviewer on the very high proportions of unclassified lineages, which raise the question of the accuracy of your sequence assignation.

Since each member of a triplicate came from the same location, they are subsamples in a statistical sense and treating each as independent is not appropriate. Our intent was to describe the community response and stream condition of the entire reach (or stream) rather than each individual point. Doing otherwise would have been a large scale mismatch because the MBSS benthic macroinvertebrate samples are also pooled from several subsamples throughout the same reach. Also, pooling the triplicates places the sample farther out on the species-area curve and collects more information about the entire community than a single member of the triplicate could.

Regarding OTU classification percentages. Low classification is not uncommon for 16S rRNA data from streams and was disappointing, but expected as we only used a single database. Simonin et al. (2019; Environmental Microbiology) also had low numbers (~940 OTUs) from stream sediments, but they did not list classification percentages. We believe that while the observation/criticism is correct, the point is not pertinent for this study. The analysis is agnostic of identity at the OTU level, and all OTUs (even unclassified) were used in the DAPC analysis at the OTU-level as stated in the methods. The fact that the higher levels (order, family, genus) often performed better on validation data highlights that aggregation to higher taxonomic level was better than focusing on all OTUs. We have added this point to the Discussion **Paragraph beginning Line 519 and Lines 651-653** to add more clarity. I have copied this same response later in this document where it was also raised.

In addition to the comments of the reviewer, I have also some comments and suggestions about  you manuscript.

1. I regret that absolutely nothing is provided at the beginning of the results on the composition and structure of your microbial communities. Moreover, it would have been interesting to see for example if there were change in the richness/diversity of these communities depending on the ecological status of the sampling sites.

We have addressed this in the results section in two ways. First, after a brief listing of dominant phyla and reference to **S Figure 1** containing the information, we explain (Line358) that an in-depth analysis of community richness and diversity of the data in the context of landscape setting is found in the companion paper, Laperriere et al. 2020.

Secondly, (**Paragraph beginning Line 463**) we provide more detailed information on the community as well as in the **Paragraph beginning Line 536** in the Discussion.

Our intent in this paper is to show that 16S data can be used to predict ecological condition, not to describe basic community structures and properties. We hope that this provides enough background for the reader to have an idea of the overall community structure.

2. I'm very surprising to see that the best results were obtained in your stream assessment classification when considering the order level and also that the depending of the nature of the samples (sediment versus water, suumer versus spring), species, genus or order provide the best result. IThis point need to be discussed.

We have expanded our discussion of these results in the **Paragraph beginning Line 519** and in a different context in the **Paragraph beginning Line 551**.

3. I don't have understood why you choose to look at the Community turnover by taking into account DAPC analysis showing high classification accuracies at the order level knowing that there were less accurate that at genus or species levels?

The order-level was, on balance, more accurate than either the genus or species when accounting for the validation data and fuzzy classification. A training set can be easily overfit to yield high accuracy, but not really that good at predicting to new cases. This is why we predicted to the validation set, and more importantly, the fuzzy classification of the validation set to allow for a better representation of a categorical response that really exists in a continuous space. We decided to use order level for community turnover precisely because it worked best for the DAPC (please see Figure 3c). Our intention was to examine community turnover at the taxonomic level that best worked for predicting the stream condition via the BIBI.

We have added some text to this extent in the **Discussion beginning Line 522**.

4. Figure 6 is quite impossible to read. Thnaks to remove the legend from the figure and to change the format of these legends (why o__RB41  and not only RB4?)

This was an oversight on my part, and I regret any confusion or frustration. The prefix has been removed and the legend shortened. There is still a single line for one OTU that crosses part of the legend, but does not impede the figure. We feel the legend is important to retain as it identifies the strongest responders. To make things more legible, I have used a single instance of the relationship for example purposes (pH) along with the entire community response in a bottom panel to more effectively demonstrate the notion of a relatively smooth community response despite specific taxa showing step function responses. I moved the remainder of the original panels into supplementary materials. Because I reference other relationships, I have included additional supplementary panel plots for taxon-environment responses.

5. I found that like for the result part, you must more deeply discussed your microbiological data. For example, nothing is provided on the orders being important in DAPC model knowing numerous papers have been published int he 10 past years on the comparison of microbial communities in more or less polluted trerrestrial or aquatic ecosystems.

We have added some natural history information for some of the important orders in the **Discussion Paragraph beginning 536** as requested. A difficulty, noted in the discussion, is that many orders are quite functionally diverse and it is difficult to generalize for every order, but we tried.

**Journal Requirements:**

1. When submitting your revision, we need you to address these additional requirements.

Please ensure that your manuscript meets PLOS ONE's style requirements, including those for file naming. The PLOS ONE style templates can be found at
http://www.journals.plos.org/plosone/s/file?id=wjVg/PLOSOne_formatting_sample_main_body.pdf and <http://www.journals.plos.org/plosone/s/file?id=ba62/PLOSOne_formatting_sample_title_authors_affiliations.pdf>

I have checked the formatting to the best of my abilities and attention span.

2. We note that you have stated that you will provide repository information for your data at acceptance. Should your manuscript be accepted for publication, we will hold it until you provide the relevant accession numbers or DOIs necessary to access your data. If you wish to make changes to your Data Availability statement, please describe these changes in your cover letter and we will update your Data Availability statement to reflect the information you provide.

Our data availability statement and accession number are in **Line 242** of the revision.

3. In your Methods section, please provide additional information regarding the permits you obtained for the work. Please ensure you have included the full name of the authority that approved the field site access and, if no permits were required, a brief statement explaining why."""

The requested information was included beginning on **Line 170**.

4. In your methods section please include the geographic coordinates for the data set.

Bounding coordinates were added – **Line 173**

5. We note that  Figure(s) 1 in your submission contain [map/satellite] images which may be copyrighted. All PLOS content is published under the Creative Commons Attribution License (CC BY 4.0), which means that the manuscript, images, and Supporting Information files will be freely available online, and any third party is permitted to access, download, copy, distribute, and use these materials in any way, even commercially, with proper attribution. For these reasons, we cannot publish previously copyrighted maps or satellite images created using proprietary data, such as Google software (Google Maps, Street View, and Earth). For more information, see our copyright guidelines: http://journals.plos.org/plosone/s/licenses-and-copyright.
 
We require you to either (1) present written permission from the copyright holder to publish these figures specifically under the CC BY 4.0 license, or (2) remove the figures from your submission:
 
a) You may seek permission from the original copyright holder of Figure(s) [#] to publish the content specifically under the CC BY 4.0 license.  
 
We recommend that you contact the original copyright holder with the Content Permission Form (http://journals.plos.org/plosone/s/file?id=7c09/content-permission-form.pdf) and the following text:
“I request permission for the open-access journal PLOS ONE to publish XXX under the Creative Commons Attribution License (CCAL) CC BY 4.0 (http://creativecommons.org/licenses/by/4.0/). Please be aware that this license allows unrestricted use and distribution, even commercially, by third parties. Please reply and provide explicit written permission to publish XXX under a CC BY license and complete the attached form.”
 
Please upload the completed Content Permission Form or other proof of granted permissions as an "Other" file with your submission.
 
In the figure caption of the copyrighted figure, please include the following text: “Reprinted from [ref] under a CC BY license, with permission from [name of publisher], original copyright [original copyright year].”
 
b)  If you are unable to obtain permission from the original copyright holder to publish these figures under the CC BY 4.0 license or if the copyright holder’s requirements are incompatible with the CC BY 4.0 license, please either i) remove the figure or ii) supply a replacement figure that complies with the CC BY 4.0 license. Please check copyright information on all replacement figures and update the figure caption with source information. If applicable, please specify in the figure caption text when a figure is similar but not identical to the original image and is therefore for illustrative purposes only.
The following resources for replacing copyrighted map figures may be helpful:
 
USGS National Map Viewer (public domain): http://viewer.nationalmap.gov/viewer/
The Gateway to Astronaut Photography of Earth (public domain): http://eol.jsc.nasa.gov/sseop/clickmap/
Maps at the CIA (public domain): https://www.cia.gov/library/publications/the-world-factbook/index.html and https://www.cia.gov/library/publications/cia-maps-publications/index.html
NASA Earth Observatory (public domain): http://earthobservatory.nasa.gov/
Landsat: http://landsat.visibleearth.nasa.gov/
USGS EROS (Earth Resources Observatory and Science (EROS) Center) (public domain): http://eros.usgs.gov/#
Natural Earth (public domain): http://www.naturalearthdata.com/

The figure has been revised to use only US State and Federal government supplied boundaries. The USA and Maryland outlines come from the US Census Bureau (https://catalog.data.gov/dataset/tiger-line-shapefile-2017-nation-u-s-current-state-and-equivalent-national), and the water boundary comes from the Maryland Geological Survey (<https://data.imap.maryland.gov/datasets/e8c38f75ba9b483ab4e668c86e33a1fa_0>).

I have added credit to both in the figure caption.

**Comments to the Author**

5. Review Comments to the Author

Please use the space provided to explain your answers to the questions above. You may also include additional comments for the author, including concerns about dual publication, research ethics, or publication ethics. (Please upload your review as an attachment if it exceeds 20,000 characters)

Reviewer #1: Revision on PONE-D-19-23872. This manuscript proposes the microbiome as tool to characterize the ecological condition of freshwater streams after their results comparison with more traditional biotic integrity indexes based on macroinvertebrates. In addition, modelling based on gradient forest was performed to identify the association of particular taxa with environmental conditions. Below I address some questions/suggestions aiming to improve this manuscript.

This study proposes the microbiome as tool to assess the level of disturbance and degradation of freshwater streams. In L 523-526, the authors highlight that the ubiquity and short generation times of microorganisms would permit a more closely track of environmental conditions comparing to macrofauna. However, the proved adaptability of microbes (ex. gene transfer confering resistance) to a range of perturbations is never discussed.

While the referee is correct about the adaptability, we felt that this would detract more than aid the manuscript. Our reason is because we found numerous indicator taxa as well as that microbes could predict stream condition. These relationships would not exist if the microbes were so flexible to adapt to all such conditions and there would therefore be no response to gradients or to different stream conditions.

L 128-138. Discriminant Analysis of Principal Components (DAPC) is mostly used in population genetics were sequences are phylogenetically close (same species). To which extent (provide descriptor) this methodological approach is robust in your analysis of community genetics? This need to be discussed in the methods section.

I have added some description of how our approach is equivalent to the SNP analysis used in population genetics. The text occurs beginning **Line 271**.

Authors never explain how do they switch from analysing bacteria and archaea separately (Table 2) to mix all together (Figures 2 to 5). Please, explain somewhere in the methods the rationale behind.

We did not analyze bacteria and archaea separately. We provided a breakdown of the richness contributions of both groups in Table 2 for the reader, but never dealt with them separately for analyses.

L214-216. Why the authors pooled triplicates samples of DNA extraction in sediments? Keeping replicates separately could have helped to correct the imbalance between sites having very poor scores (n = 5) and good scores (n = 37). How this imbalance can further influence in DAPC and GA analyses?

We pooled before sequencing, and the purpose was to get as broad a representation as possible from a reach before sequencing. Our sampling unit was the stream reach, and therefore each replicate was not a replicate, but a subsample. There was no loss of power.

Did you perform DAPC (and further statistical analyses) with even sequence depths among samples? Did you run rarefaction curves prior to DAPC? Were these sequences selected at random?

The data were normalized to 1344 sequences. This was written in the next to last sentence of the “Bioinformatic Analysis” section (**Line 261** current version), but was probably missed by the referee

Accession number of 16S rRNA sequencing results must be supplied in the manuscript.

We have included the accession number (PRJNA545742) on **Line 242**.

The extremely low affiliation of OTUs sequences into species (ex. 593 classified against 59438 unclassified) is normal when using this kind of sequencing approach? If this is an optimal result, authors should highlight this in the discussion section. If not, the sequencing analysis should be re-done.

Regarding OTU classification percentages. Low classification is not uncommon for 16S rRNA data from streams and was disappointing, but expected as we only used a single database. Simonin et al. (2019; Environmental Microbiology) also had low numbers (~940 OTUs) from stream sediments, but they did not list classification percentages. We believe that while the observation/criticism is correct, the point is not pertinent for this study. The analysis is agnostic of identity at the OTU level, and all OTUs (even unclassified) were used in the DAPC analysis at the OTU-level as stated in the methods. The fact that the higher levels (order, family, genus) often performed better on validation data highlights that aggregation to higher taxonomic level was better than focusing on all OTUs. We have added this point to the Discussion **Paragraph beginning Line 519** and **Lines 651-653** to add more clarity.

Threshold values considered as acceptable for % accuracy (Figures 2 and 3) and/or R2 (Figure 5) are never discussed in the manuscript. Please provide information in the methods section.

We used no formal threshold as any value would be difficult to justify. Rather, we judged performance based on the among-group differences. Any approach using environmental data, with all of its noise, that has an accuracy above 70% is a pretty good result. However, all of the DAPC analyses were subject to the Kappa statistic to test departure from randomness, and as stated in the results, all of the comparisons mentioned as good performers were significantly better than random assignments, even after correcting for differences in the uneven distribution of BIBI scores. Regarding Figure 5, we again used judgement and cut things off where the R2 fell off steeply.

According to Figure 6 results, DOC and carbon had a strong weight in explaining important taxa distribution in spring. However, co-variation between these two environmental variables tends to occur in samples of water. Authors should should these kind of problems in their analysis.

The percent carbon was calculated only for sediment samples. I have added this to the description in Table 1. This was an oversight on my part. Sorry for any confusion. Interestingly, there was little overlap in the taxa responding strongly to these variables.

L 478. Many studies have already worked on the biogeographic distribution of microorganisms. A deeper comparison between these studies and the results obtained in the present study would be needed in the discussion section.

We have expanded on this in the discussion in multiple places. **Lines 537-545, 565, 587**.

L 571-577. The authors should precise that in the case of “functional approaches” metatranscriptomic analyses would be more appropriate than metagenomics analyses.

Point taken and revised accordingly to reflect ecosystem functions and not specifically metabolic products. **Line 657**.

Minor comments

L39-40. At this step the terms “training data” and “validation data” have not yet been defined. This can difficult the reader understands.
Descriptors added in the abstract, **Line 40**.

L43-45. This sentence is a bit contradictory to me since the aim of high throughput sequencing is to enhance the depth of analyses in microbial diversity. Rewrite.
I have revised the sentence, but the referee is missing the point that while high throughput sequencing allows us to do the initial modeling, the subset allows us to predict condition without having to redo the modeling each and every time a new sample is collected.

L97-98. This statement is not true for micro-eukaryotes such as diatoms. Correct.
Corrected to suggest that most follow this pattern. **Line 95**.

L178. Were water samples collected in triplicate?
No, because the water sample was a much larger volume than the sediment cores.

L180. What is the “C1” solution? Please describe.
We have described this in the methods. **Line 185**

L181-182. Did you use a cell disruptor? Please, provide exact speed values. Idem for L 192.

The information was in the original, but must have been missed by the referee. The requested information is in the first paragraph of the “DNA extraction and library preparation” subsection.

L 222. Not clear to which primers you refer here. Precise.

We have added the link to the documentation for the Nextara primers immediately following the referenced sentence on **Line 231**.

L344. Accuracy values for Fig 2a are even lower than 43% (ex. genus and phylum level). Correct.

That’s embarrassing – corrected. Thanks for noticing that. **Line 367**.

The authors decided to present a selection of their results (ex. Figure 5 and 6) that in my opinion could be placed as supplementary information.

We have moved the original Figure 5 into **S2 Figure**. Similarly, we have revised the original Figure 6 to better reflect our point and moved the panel plot into **S3-5 Figures** as well.

Please correct typing errors on SI units everywhere in the manuscript and especially in the methods section.

 Done – I think I found them all.

While revising your submission, please upload your figure files to the Preflight Analysis and Conversion Engine (PACE) digital diagnostic tool, <https://pacev2.apexcovantage.com/>. PACE helps ensure that figures meet PLOS requirements. To use PACE, you must first register as a user. Registration is free. Then, login and navigate to the UPLOAD tab, where you will find detailed instructions on how to use the tool. If you encounter any issues or have any questions when using PACE, please email us at [figures@plos.org](mailto:figures@plos.org). Please note that Supporting Information files do not need this step.

All figures and supporting figures were uploaded, checked, and passed the PACE tool.
