# Supplementary material for: Microbial communities can predict the ecological condition of headwater streams
Source: PLoS One. 2020 Aug 3;15(8):e0236932. doi: 10.1371/journal.pone.0236932 (PMC7398514; doi:10.1371/journal.pone.0236932)
Supplement: Attachment — Submitted filename: response to reviewers.docx [file pone.0236932.s007.docx]

I have addressed the two remaining comments that were requested after the second round of reviews. I have included the review comments below, and I have highlighted my responses in yellow as well as indicated where those changes occur in the revision in **bold**.

I hope that my revision is suitable for acceptance and publication in PLOS ONE. Thank you. bob

####################

6. Review Comments to the Author

Please use the space provided to explain your answers to the questions above. You may also include additional comments for the author, including concerns about dual publication, research ethics, or publication ethics. (Please upload your review as an attachment if it exceeds 20,000 characters)

Reviewer #1: The authors have consistently improved their manuscript. All questions I addressed have been answered and/or taken into account in the revised manuscript. However, still one important aspect remains unaddressed in the revised MS.

Original question from the reviewer. L214-216. Why the authors pooled triplicates samples of DNA extraction in sediments? Keeping replicates separately could have helped to correct the imbalance between sites having very poor scores (n = 5) and good scores (n = 37).

Answer from the authors. We pooled before sequencing, and the purpose was to get as broad a representation as possible from a reach before sequencing. Our sampling unit was the stream reach, and therefore each replicate was not a replicate, but a subsample. There was no loss of power.

I understand the point of view of the authors. However, their choice of pooling PCR amplicons needs to be justified in the manuscript. The explanation you gave above would be pertinent according to your dataset “Doing otherwise would have been a large scale mismatch because the MBSS benthic macroinvertebrate samples are also pooled from several subsamples throughout the same reach”.

I have added the requested text – **lines 227-230**.

But further, the overall message of the MS is that monitoring of microbial communities in sediments can be considered as a complementary tool to assess the ecological condition of streams. Then, you need to precise how sediment samples were exaclty taken (in terms of granulometry, centre/edge of stream cannel...among others) to guide other researchers when applying your strategy. As you certainly know bacterial functions and diversity in stream sediments can change drastically depending of the conditions cited above. These aspects needs to be clearly defined in the M&M section of your MS.

I have added detail to the sediment sampling on **lines 171-174**.
